# Use of a Radiomics Model to Predict Tumor Invasiveness of Pulmonary Adenocarcinomas Appearing as Pulmonary Ground-Glass Nodules

**DOI:** 10.1155/2018/6803971

**Published:** 2018-06-13

**Authors:** Xing Xue, Yong Yang, Qiang Huang, Feng Cui, Yuqing Lian, Siying Zhang, Linpeng Yao, Wei Peng, Xin Li, Peipei Pang, Jianhua Yan, Feng Chen

**Affiliations:** ^1^Department of Radiology, The First Affiliated Hospital, College of Medicine, Zhejiang University, China; ^2^Department of Thoracic Surgery, Hangzhou Hospital of Traditional Chinese Medicine, China; ^3^Department of Radiology, Hangzhou Hospital of Traditional Chinese Medicine, China; ^4^Department of Pathology, The First Affiliated Hospital, College of Medicine, Zhejiang University, China; ^5^GE Healthcare, China; ^6^Department of Radiology, The Second Affiliated Hospital of Wenzhou Medical University, Wenzhou, China

## Abstract

**Background:**

It is important to distinguish the classification of lung adenocarcinoma. A radiomics model was developed to predict tumor invasiveness using quantitative and qualitative features of pulmonary ground-glass nodules (GGNs) on chest CT.

**Materials and Methods:**

A total of 599 GGNs [including 202 preinvasive lesions and 397 minimally invasive and invasive pulmonary adenocarcinomas (IPAs)] were evaluated using univariate, multivariate, and logistic regression analyses to construct a radiomics model that predicted invasiveness of GGNs. In primary cohort (comprised of patients scanned from August 2012 to July 2016), preinvasive lesions were distinguished from IPAs based on pure or mixed density (PM), lesion shape, lobulated border, and pleural retraction and 35 other quantitative parameters (P <0.05) using univariate analysis. Multivariate analysis showed that PM, lobulated border, pleural retraction, age, and fractal dimension (FD) were significantly different between preinvasive lesions and IPAs. After logistic regression analysis, PM and FD were used to develop a prediction nomogram. The validation cohort was comprised of patients scanned after Jan 2016.

**Results:**

The model showed good discrimination between preinvasive lesions and IPAs with an area under curve (AUC) of 0.76 [95% CI: 0.71 to 0.80] in ROC curve for the primary cohort. The nomogram also demonstrated good discrimination in the validation cohort with an AUC of 0.79 [95% CI: 0.71 to 0.88].

**Conclusions:**

For GGNs, PM, lobulated border, pleural retraction, age, and FD were features discriminating preinvasive lesions from IPAs. The radiomics model based upon PM and FD may predict the invasiveness of pulmonary adenocarcinomas appearing as GGNs.

## 1. Introduction

The detection rate of pulmonary nodules has increased due to advances in diagnostic imaging and the widespread use of low-dose chest CT screening. Since many nodules characterized as ground-glass nodules (GGNs) have a higher likelihood of malignancy [1, 2], GGNs are receiving more attention on routine chest CT scans.

The GGN is defined as a hazy increased density on lung windows with abnormal vascularity and the presence of the air bronchogram sign [3]. In 2011, the International Association for the Study of Lung Cancer (IASLC), the American Thoracic Society (ATS), and the European Respiratory Society (ERS) set a new standard for the classification of lung adenocarcinoma. According to this classification, pulmonary adenocarcinomas were classified as atypical adenomatous hyperplasia (AAH), adenocarcinoma in situ (AIS), minimally invasive adenocarcinoma (MIA), and invasive adenocarcinoma (IAC) [4]. So far, there is no uniform guidance regarding the surgical procedures for GGNs. Several studies have shown that follow-up or sublobar resection is recommended in cases of AAH and AIS [5–7], because, if completely resected, patients can achieve 100% and near 100% 5-year disease-free survival (DFS) rates, respectively [8–10]. Therefore, it is important to distinguish preinvasive lesions (AIS/AAH) from invasive pulmonary adenocarcinomas (IPAs) (including MIA and IAC) before operation so that the surgeon may select suitable candidates for resection.

Several studies have focused on distinguishing the pathological types of GGNs [11–14]. A recent study revealed that the EGFR mutation rate in invasive adenocarcinoma was higher than the mutation rate found in other types of pulmonary adenocarcinomas classified as GGNs [11]. If enhanced imaging metrics were used, the diagnostic power of chest CT could be improved for invasive adenocarcinoma appearing as pure GGN or partially solid nodules when compared with conventional noncontrast chest CT imaging [12]. It has been demonstrated that tumor size and the proportion of solid volume are significant predictors of pathological invasiveness in mixed nodules [13]. Some studies have also shown that CT attenuation values and entropy were helpful in distinguishing invasive adenocarcinoma from preinvasive or MIA [14]. However, these studies were limited by either small numbers of patients or few quantitative features that were investigated.

The present study had as its aim enrolling a relatively large number of patients with approximately 599 GGNs in order to distinguish between preinvasive versus invasive lesions using a radiomics model based on both qualitative and quantitative features of GGNs on chest CT.

## 2. Materials and Methods

This study was approved by our Institutional Review Board. From August 2012 to July 2016, records from patients with pathologically confirmed lung adenocarcinoma were retrospectively reviewed. CT reports were searched for target patients by using the keywords “GGN”, “ground glass opacity”, “part-solid nodule”, and “ground-glass”.

The inclusion criteria for the study were as follows: (1) patients were scanned with routine chest CT using a slice thickness of 5.0 mm; (2) diagnosis without distant metastasis was confirmed by surgery and pathology; (3) only the last CT scan before surgery was chosen for the study; (4) nodules with pure GGNs or a partially solid component were selected on CT scans; and (5) the diameter of each nodule was between 5.0 and 30.0 mm.

All patients were placed in supine position on the scanning bed and were instructed to take a deep inspiration and hold their breath during the chest CT scan. The mean interval of time between the CT scan and surgery was 8.72 ± 9.06 days. Three CT scanners were used (Toshiba Aquilion 16, Toshiba Medical Systems, Tokyo, Japan; Brilliance 64 and MX8000, Philips Medical Systems, Best, The Netherlands) in the study. The scan parameters included the following: 120 kVp, 100-200 mAs, and 5.0 mm collimation. Images were reconstructed using a medium sharp reconstruction algorithm with a slice thickness of 5.0 mm. Patients who were hospitalized before Jan 2016 were assigned to the primary cohort and patients hospitalized after Jan 2016 were assigned to the validation cohort.

### 2.1. Analysis of Features

The clinical data collected for each patient included their age, gender, smoking status, lesion location, chest symptoms, and medical history.

Nodule morphology was assessed through discussion by two chest radiologists with 3- and 10- year experience, respectively, with consensus. The CT findings were analyzed on the lung window setting with a window level of 1500 HU and a window width of -430 HU in a picture archiving system.

The following morphologic characteristics of GGNs were estimated and recorded: (a) pure GGNs (pGGNs) or mixed GGNs (mGGNs). pGGNs were defined as nodules with no solid component whereas mGGNs were defined as nodules with a partially solid component, and a solid component was defined as any opacity that completely obscured the lung parenchyma; (b) lesion shape: round or oval shape was defined as a regular shape whereas any other configuration was defined as irregular in shape; (c) bubble lucency: it was considered to be present when small spots of round or ovoid air attenuation were present in a GGN; (d) air bronchogram: it is defined when air-filled bronchi were seen within the GGN; (e) lobulated border: it is defined as a wavy or scalloped configuration; (f) clear margin: it is defined as the easily identifiable boundary of a nodule; (g) spiculated sign: it is defined as the presence of strands extending from the nodule margin into the lung parenchyma; (h) pleural retraction: it was defined by the proximity of the pleural to the GGN; (i) abnormal blood vessels: they are defined as the vessels collecting inside the tumor, passing through the lesion, or interrupting the lesion.

Quantitative features were analyzed by software developed by the authors (AK, GE healthcare, US), as follows. A nodule was first segmented by drawing a region of interest (ROI) covering as large an area as possible from all the slices containing the nodule. The ROI was drawn in freehand by two observers using an electronic mouse, avoiding large vessels and bronchi. After segmentation, quantitative two-dimensional textural features were calculated and extracted automatically by the software. An average of the values from two observers was used as the final result.

### 2.2. Statistical Analysis

All features noted in both preinvasive lesions and IPAs were compared using the student t-test and chi-square test. Clinical data, morphologic characteristics, and texture of GGNs in the primary cohort were compared using univariate analysis and multivariate analysis to determine the association between preinvasive adenocarcinomas and IPAs. The effects of multiple factors on a nomogram were determined by logistic regression analysis, and only the factors that reached significance were incorporated into the nomogram. The receiver operating characteristic (ROC) curve was used to evaluate the efficacy of the final model. All analyses were performed using the SPSS version 20.0 software program. A P value < .05 was considered statistically significant.

## 3. Results

A total of 570 patients with 599 GGNs were enrolled in the study. Out of 570 patients, 403 patients were female with 428 GGNs, and 167 patients were male with 171 GGNs. The mean age of the patients was 58.37 ± 11.40 years (range, 18-81 years).

There is no statistically significant difference of sexes between the preinvasive and invasive lesions (P=0.942). The 599 GGNs consisted of 397 IPAs (66.3 %), including MIAs and IACs, and 202 preinvasive lesions (33.6%). Of the 570 patients, 37 patients each had two GGNs and one patient had three GGNs. In the primary cohort, there were 453 patients with 484 GGNs, and in the validation cohort there were 107 patients with 115 GGNs (Table 1).

### 3.1. Univariate and Multivariate Analysis

The characteristics of the two cohorts are summarized in Tables 2 and 3. According to univariate analysis (Table 2), pure or mixed lesions (PM) (P <0.001), lesion shape (P =0.050), lobulated border (P =0.001), presence of pleural indentation (P <0.001), age (P =0.003), fractal dimension (FD) (P <0.001), and most of the textural analysis (see Table 2 for further details) were significantly associated with invasive extent of the GGNs.

**Figure 1:**
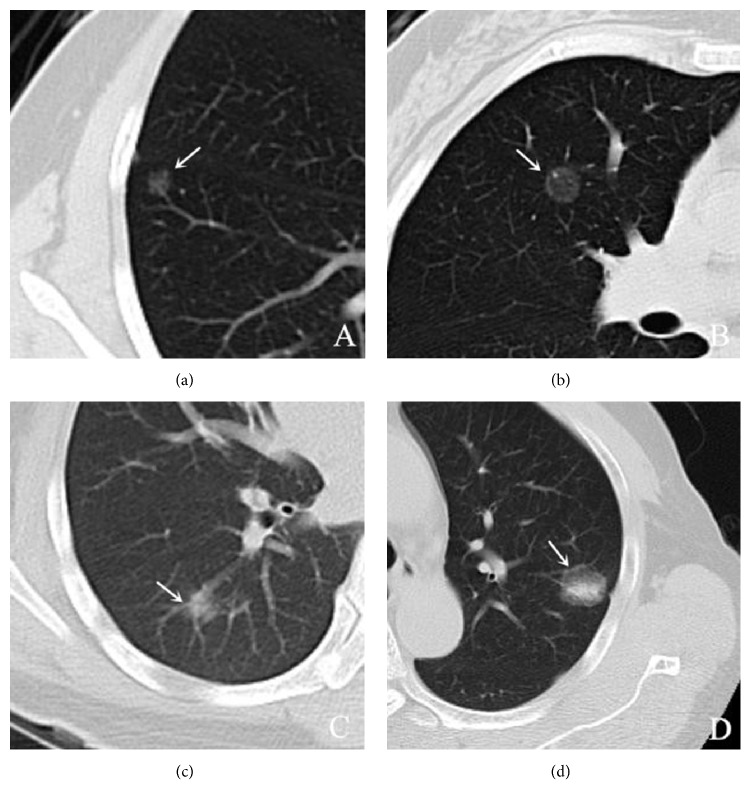
CT images show preinvasive lesions and invasive adenocarcinomas. (a) A 7 mm mGGN (arrow) is shown in the anterior segment of the right upper lobe in a 53-year-old woman. This nodule was confirmed pathologically as a preinvasive lesion (AAH). (b) A 13 mm mGGN (arrow) is shown in the anterior segment of the right upper lobe in a 53-year-old woman. This nodule was confirmed pathologically as a preinvasive lesion (AIS). (c) An 11 mm mGGN (arrow) is shown in the posterior segment of the right upper lobe in a 35-year-old woman. This nodule was confirmed pathologically as an invasive pulmonary adenocarcinoma (MIA). (d) A 21 mm mGGN (arrow) is shown in the posterior segment of the right upper lobe in a 35-year-old woman. This nodule was confirmed pathologically as an invasive pulmonary adenocarcinoma (IAC). Note: AAH=atypical adenomatous hyperplasia; AIS=adenocarcinoma in situ; MIA=minimally invasive adenocarcinoma; IAC=invasive adenocarcinoma.

On multivariate analysis, five features including PM (P <0.001), lobulated border (P =0.040), presence of pleural indentation (P =0.002), age (P =0.020), and FD (P <0.001) were found to be significant in differentiating between preinvasive lesions and IPAs (Figure 1, Table 3).

### 3.2. Construction and Performance of Radiomics Nomogram

**Figure 2:**
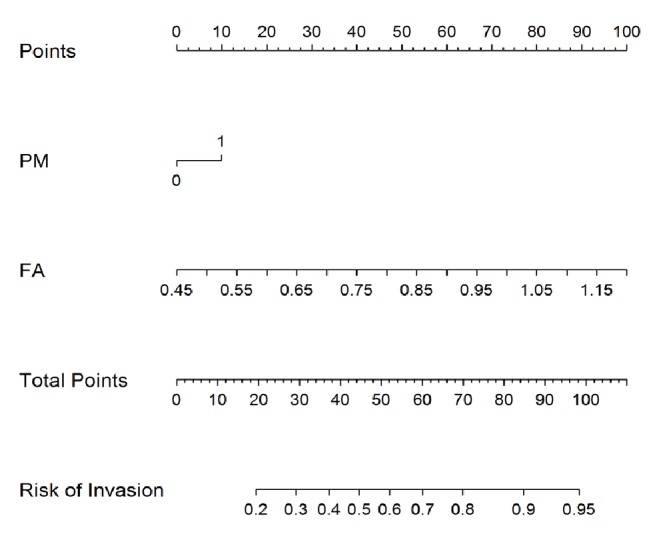
**A nomogram to predict the invasiveness of GGNs**. The nomogram is based on the radiomic features of pure or mixed lesions (PM) and fractal dimension (FD) of the primary cohort. The probability of each GGN value is marked on each axis. The FD value of the preinvasive lesions in the validation cohort was 0.79 ± 0.11, and the FD value of the IPAs in the validation cohort was 0.94 ± 0.17.

**Figure 3:**
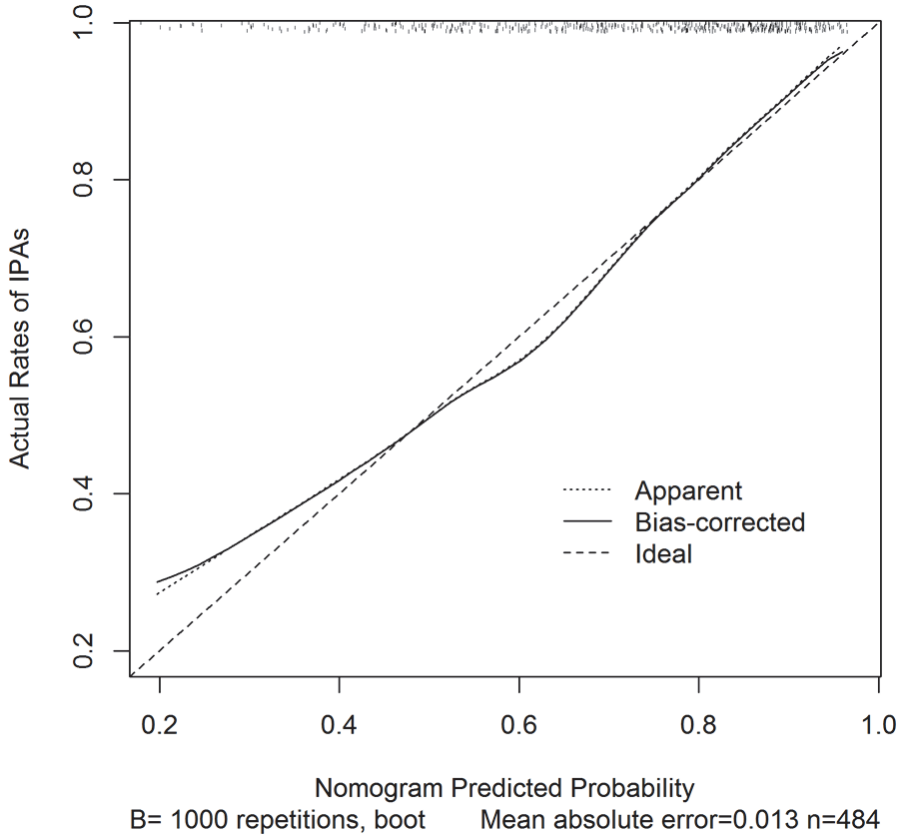
**Calibration plot of the relationship between predicted and actual rates of invasive pulmonary adenocarcinomas (IPAs) in the validation cohort. **The x-axis represents the prediction from the radiomic nomogram, and the y-axis represents the actual occurrence of IPAs. The dashed line shows the ideal nomogram. The solid line indicates the performance of the nomogram applied to the validation cohort.

After logistic regression analysis, PM and FD were the features selected among the five features to build the nomogram (Figure 2). The calibration curve of the radiomics nomogram performance is shown in Figure 3. The solid line in the figure indicates the performance of the nomogram.

**Figure 4:**
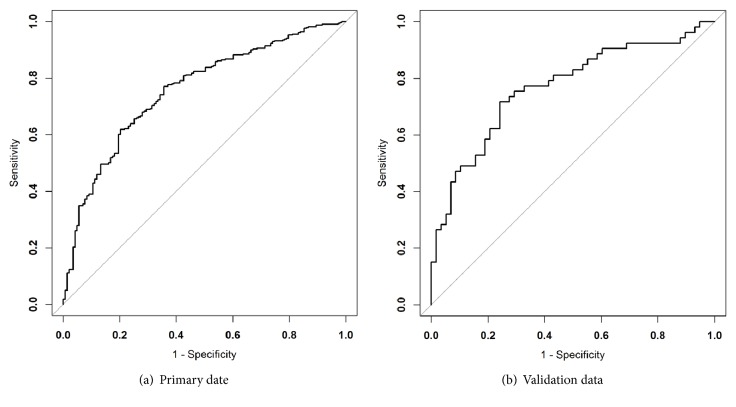
**Receiver operating characteristic curve for the prediction of the radiomic nomogram.** ROC is based on the combination of the 95th percentile pure or mixed lesions (PM) and fractal dimension (FD) and shows significant diagnostic accuracy in both primary and validation cohorts. AUC for the primary (a) and validation cohorts (b) was 0.757 (95% CI: 0.711 to 0.803) and 0.793 (95% CI: 0.708-0.877), respectively.

The nomogram calibration plot showed good predictability. The rate of predicted results paralleled the real status of IPAs, almost exactly correlating with the 45-degree line on the graph (Figure 3). The correspondence between actual and ideal nomogram predictions suggests good calibration of the nomogram in the validation cohort. ROC analysis revealed that the area under curve (AUC) for the primary and validation cohorts was 0.76 (95% CI: 0.71 to 0.80) and 0.793 (95% CI: 0.71 to 0.88), respectively (Figure 4). Comparison of these two ROCs ( 0.76 versus 0.79) yielded a P value = 0.47.

## 4. Discussion

To the best our knowledge, the present study is the first to use a quantitative radiomics model, incorporating clinical and imaging features, to differentiate preinvasive lesions from IPAs appearing as GGNs.

The major findings of this study are summarized as follows. Five features of PM lesions were found to be significant in differentiating preinvasive lesions from IPAs including PM, lobulated border, pleural indentation, age, and FD. In addition, a nomogram incorporating both the PM and FD features was further used to differentiate preinvasive lesions from IPAs. The mGGNs were found to be the predominant type in our IPA group, which is consistent with previously published studies.

Matsuguma H et al. [15] reported that the component of ground-glass opacity was inversely related to the invasive nature of GGNs. Lee SM et al. [16] found that a smaller solid proportion was a key sign in distinguishing preinvasive lesions from IPAs in partially solid GGNs. Another study, on the other hand, found that a greater amount of solid component, presence of alveolar interval thickening, and reduced gas may be more frequently seen in IPAs [17]. Some researchers showed that a lobulated border was more frequent in IPAs than preinvasive nodules [16, 18]. However, Lee SM et al. [16] demonstrated that preinvasive lesions usually have nonlobulated borders compared with IPAs. Some reports concluded that pleural indentation shown on CT was significantly associated with the extent of invasiveness of a nodule pathologically, although other researchers did not agree with that opinion [16, 19–22].

Our study confirmed the finding that pleural indentation is more frequently associated with IPAs, either in pGGNs (P =0.005) or in mGGNs (P =0.017) [16]. Our results also implied that older patients with GGNs have a higher probability of IPAs, although few studies have supported such a finding.

Fractal analysis is a mathematical construct which can be used to evaluate and quantify texture or heterogeneity of a tumor on CT images [23–25]. It is a versatile and sensitive tool that has much potential application to lung cancer [26]. As a physical measure and noninteger value, FD is considered to be related to the complexity of an object. In a previous study, Al-Kadi OS et al. [27] reported that higher FDs may be seen in a majority of late-stage lung cancers as compared with early-stage lesions. This implies that the more aggressive the tumor, the higher the FD of the tumor. In our study, the accuracy of using FD to distinguish advanced-stage from early-stage lung tumors was 83.3%. Our results also demonstrated that a higher FD was compatible with greater invasiveness of a GGN. Thus, FD may be a useful indicator for distinguishing preinvasive from invasive lesions.

Radiomics refers to a method that extracts and analyzes quantitative imaging features with high throughput, which can help to noninvasively predict tumor behavior and heterogeneity [28–30]. There are several reasons why radiomics is applicable to lung nodules. The high contrast resolution between pulmonary nodules and lung parenchyma renders pulmonary nodules ideal candidates for evaluation. Another reason involves the presence of heterogeneity within pulmonary nodules on CT, which cannot be easily observed by the naked eye. Also, a lung cancer dataset can be accessed via large interinstitutional databases, which is helpful when attempting to extract useful quantitative imaging features [31]. The radiomics nomogram [32, 33] provides estimates, based on the tumor's characteristics including qualitative and quantitative indexes, which are selected after dimensionality reduction.

In a CT texture study, Chae HD et al. [34] successfully differentiated preinvasive lesions from IPAs using smaller mass and higher kurtosis as differentiators in partially solid GGNs. In another study, She Y et al. [35] used a nomogram to identify IPAs in a set of solitary pure GGNs and concluded that lesion size, lesion margin, lesion shape, mean CT value, presence of pleural indentation, and smoking status were factors predicting invasive extent.

The present study has some divergent aspects with the above literature. Our patient group included pure and mixed GGNs, a feature that was included in our nomogram. Thus, we found that the solid component was a key indicator in predicting invasiveness of GGNs. In addition, as a novel quantitative imaging feature, FD was incorporated into our nomogram, which has not yet been reported in the radiomics nomogram literature. As a result, our model was highly successful in predicting invasiveness in GGNs in our validation cohort.

According to recent studies [11, 36], the EGFR mutation rate in the female patients was significantly higher than that of the male patients with ground-glass nodules in both western and eastern countries. That may explain why the proportion of women is higher than that of men in our study, though there is no significant difference of sexes between the preinvasive and invasive lesions. Since the relationship between sex and gene mutation is not studied in our study, we may not conclude that the female patients in our study may possibly have a higher rate in EGFR mutations than others.

Our study has a major limitation. In this study, we used a slice thickness of 5.0 mm for CT scan, which is not optimal for visualizing the small lung nodules. The reason is that the thin-section, e.g., 1.5 mm, CT data was not available for all patients due to the retrospective nature of the study in author's institute, because most of the thin-section data had been deleted from the workstation due to the limited memory space. In addition, chest CT scan with a 5.0-mm slice thickness is a common practice in many Chinese hospitals. As we have a very heavy daily patient workload, e.g., >1000 CT scans/day in author's institute, it is not realistic to scan with 1.5 mm slice thickness for all lung nodule patients as suggested by the Fleischner Society guidelines [37]. Indeed, 5 mm thickness may not reflect the authenticity of nodules due to partial volume effect. In our study, however, all the diameter of pulmonary nodules is >6mm, and each nodule has more than two slice images, which can basically reflect the morphological characteristics of the image. In addition, 102 nodules which underwent HRCT (high-resolution computed tomography) scan were not included in our study. They were all proved to be ground-glass nodules whether they were scanned with a thickness of 1 mm or 5 mm, indicating that a relatively bigger thickness of 5 mm CT scan may not have a great effect on our results. Therefore, we consider that taking the advantage of existing CT data with 5.0 mm slice thickness is still useful for the evaluation of GGN patients in terms of saving resources. The present study may have provided some evidences for the argument.

In conclusion, the radiomics nomogram incorporating both the features of PM and FD were able to discriminate preinvasive adenocarcinomas from IPAs. The presence of a solid component and a higher FD may indicate a higher probability of IPAs in GGNs. Thus, our results may be helpful in the clinical management of GGNs.

## Figures and Tables

**Table 1:** Characteristics of patients for preinvasive lesions and IPAs.

	All GGNs	Preinvasive lesions	IPAs	P*∗* value
	(n=599)	(n=201)	(n=398)	
Sex				
M	171(28.5%)	57(28.4%)	114(28.6%)	0.942
F	428(71.5%)	144(71.6%)	284(71.4%)	
Age	58.37±11.40	57.39±12.08	58.86±11.03	
Smoking				0.137
yes	86(14.4%)	23(11.4%)	63(15.8%)	0.148
no	513(85.6%)	178(88.6%)	335(84.2%)	
Symptom				
yes	112(18.7%)	76(19.1%)	36(17.9%)	0.725
no	487(81.3%)	322(80.9%)	165(82.1%)	
History of tumor				
yes	44(7.2%)	17(8.5%)	27(6.5%)	0.393
no	555(92.8%)	184(91.5%)	371(935%)	
Location				
Right upper	248(41.4%)	94(46.8%)	154(38.7%)	0.445
Right middle	48(8.0%)	14(7.0%)	34(8.5%)	
Right lower	85(14.2%)	25(12.4%)	60(15.1%)	
Left upper	145(24.2%)	45(22.4%)	100(25.1%)	
Left lower	73(12.2%)	23(11.4%)	50(12.6%)	
Surgery				
wedge resection	312(52.1%)	126(62.7%)	186(46.7%)	<0.01
segmentectomy	92(15.4%)	26(12.9%)	66(16.6%	
lobectomy	195(32.5%)	49(24.4%)	146(36.7)	
Density				
pure	282(47.1%)	125(62.2%)	157(39.4%)	<0.01
mixed	317(52.9%)	76(37.8%)	241(60.6%)	
Shape				
Round or oval	363(60.6%)	150(74.6%)	213(53.5%)	<0.01
irregular	236(39.4%)	51(25.4%)	185(46.5%)	
Bubble sign				
yes	139(23.2%)	39(19.4%)	100(25.1%)	0.117
no	460(78.8%)	162(80.6%)	298(74.9%)	
Air-bronchogram sign				
yes	120(20.0%)	21(10.4%)	99(24.9%)	<0.01
no	479(80.0%)	180(89.6%)	299(75.1%)	
Lobulated border				
yes	223(37.2%)	26(12.9%)	197(49.5%)	<0.01
no	376(62.8%)	175(87.1%)	201(50.5%)	
Spiculation sign				
yes	94(15.7%)	19(9.5%)	75(18.8%)	<0.01
no	505(84.3%)	182(90.5%)	323(81.2%)	
Clear margin				
yes	527(88.0%)	171(85.1%)	356(89.4%)	0.120
no	72(12.0%)	30(14.9%)	42(10.6%)	
Pleural retraction				
yes	181(30.2%)	34(16.9%)	147(36.9%)	<0.01
no	418(69.8%)	167(83.1%)	251(63.1%)	
Abnormal vessels				
yes	302(50.4%)	88(43.8%)	214(53.8%)	0.021
no	297(49.6%)	113(56.2%)	184(46.2%)	
Pathology				
AAH	80 (13.3%)	29 (14.4%)	51 (12.8%)	0.567
AIS	121 (20.2%)	38 (18.9%)	83 (20.9%)	
MIA	174 (29.2%)	65 (32.8%)	109 (27.4%)	
IAC	224 (37.3%)	69 (33.9%)	155 (38.9%)	

*∗* means P value is derived from the chi-square test and Student's t-test between preinvasive lesions and IPAs.

Note: AAH=atypical adenomatous hyperplasia, AIS=adenocarcinoma in situ, MIA=minimally invasive adenocarcinoma, IAC=invasive adenocarcinoma, and IPAs=invasive pulmonary adenocarcinomas.

**Table 2:** One-variate analysis for differentiating preinvasive lesions from IPAs.

Feature	P value
PM	0.0000446
Shape	0.05
Lobulated	0.001
Pleural	0.00003422
Age	0.003
FD	2.20E-16
FOS_entropy	0.001
FOS_ninetyfive	2.20E-16
GLCM_contrast1	0.00006972
GLCM_contrast2	2.83E-10
GLCM_dissimilarity	2.83E-10
GLCM_entropy	0.00009698
GLCM_homogeneity	2.19E-08
GLCM_idm	0.00002345
GLCM_intensity	9.10E-10
RLM_HIRE	0.004
RLM_HISRE	0.001
RLM_IV	0.001
RLM_LILRE	0.000001362
RLM_LISRE	0.0009
RLM_LRE	0.03
RLM_RLV	2.03E-14
RLM_RP	0.03
NGTD_Complexity	2.26E-07
NGTD_Contrast	0.00001651
normGLCM_contrast_1	0.0000161
normGLCM_dissimilarity	0.0000161
normGLCM_entropy	0.0001
normGLCM_intensity	0.02
NGLD_EN	0.001
NGLD_NNU	2.20E-16
NGLD_SM	0.0163
ISZ_HIZE	0.0004
ISZ_HISZE	0.001
ISZ_IV	3.16E-08
ISZ_LILZE	0.04
ISZ_LIZE	0.0014
ISZ_LISZE	0.001
ISZ_SZV	6.29E-16
ISZ_SZE	0.00005694

Note: IPAs=invasive pulmonary adenocarcinomas, PM=pure or mixed density, and FD=fractal dimension.

**Table 3:** Multivariate analysis for differentiating preinvasive lesions from IPAs.

Feature	P value
PM	7.75E-06
Lobulated sign	0.046571
Pleural retraction	0.002485
Age	0.021143
FD	1.10E-14

Note: IPAs=invasive pulmonary adenocarcinomas, PM=pure or mixed density, and FD=fractal dimension.

## Data Availability

The nature of the data is that it is part of the raw data of in-patients of the First Affiliated Hospital of Zhejiang University. The data is stored permanently in servers of the hospital. If data access is requested, an application letter can be sent to author's hospital and the data can be accessed after the application has been approved. The reason for data access restriction is that it involves patient's privacy and the safety policy of the hospital.

## Abbreviations

AAH: Atypical adenomatous hyperplasia
AIS: Adenocarcinoma in situ
ATS: American Thoracic Society
AUC: Area under curve
CI: Confidence interval
DFS: Disease-free survival
ERS: European Respiratory Society
FA: Fractal analysis
FD: Fractal dimension
GGN: Ground-glass nodule
HRCT: High-resolution computed tomography
IAC: Invasive adenocarcinoma
IASLC: International Association for the Study of Lung Cancer
IPAs: Invasive pulmonary adenocarcinomas
mGGN: Mixed ground-glass nodule
MIA: Minimally invasive adenocarcinoma
pGGN: Pure ground-glass nodule
PM: Pure or mixed lesions
ROC: Receiver operating characteristic.
